# Effect of tissue tension on magnetic compression anastomosis of digestive tract

**DOI:** 10.1038/s41598-024-65160-8

**Published:** 2024-06-20

**Authors:** Miaomiao Zhang, Jia Ma, Aihua Shi, Ruimin Gong, Xuhe Zhao, Qiuye Zhong, Linxin Shen, Yi Lyu, Xiaopeng Yan

**Affiliations:** 1 Department of Hepatobiliary Surgery, The First Affiliated Hospital of Xi’an Jiaotong University, No. 277 West Yanta Road, Xi’an, 710061 Shaanxi China; 2 Shaanxi Provincial Key Laboratory of Magnetic Medicine, The First Affiliated Hospital of Xi’an Jiaotong University, No. 277 West Yanta Road, Xi’an, 710061 Shaanxi China; 3 National and Local Joint Engineering Research Center of Precision Surgery & Regenerative Medicine, The First Affiliated Hospital of Xi’an Jiaotong University, No. 277 West Yanta Road, Xi’an, 710061 Shaanxi China; 4 Department of Surgical Oncology, Shaanxi Provincial People’s Hospital, Xi’an, China; 5 Zonglian College, Xi’an Jiaotong University, Xi’an, China

**Keywords:** Magnetosurgery, Magnetic compression anastomosis, Magnamosis, Tissue tension, Digestive tract, Gastrointestinal system, Gastrointestinal diseases, Gastroenterology

## Abstract

With the increasing application of magnetic compression anastomosis (MCA) in gastrointestinal anastomosis, we identified an interesting phenomenon that an anastomosis is more prone to stenosis after endoscopic gastrointestinal MCA. We hypothesized that the increase in tissue tension during endoscopic procedures is the cause of anastomotic stenosis. In this study, we investigated the effect of tissue tension on gastroduodenal bypass MCA in Sprague–Dawley (SD) rats. Twenty SD rats were divided into the study group (high-tension group, n = 10) and control group (no tension group, n = 10), wherein the rats underwent complete gastroduodenal bypass magnetic anastomosis under high tension and no tension of the digestive tract, respectively. Anastomotic specimens were obtained 4 weeks after the operation, and anastomotic diameters of the two groups were observed and measured. The histological difference was observed by hematoxylin & eosin and Masson staining. The operation was successfully completed in all rats, and all survived until 4 weeks postoperatively. Anastomotic measurements revealed that the anastomosis diameter was significantly smaller in the study group than in the control group, and there were three cases of severe anastomotic stenosis. Histological observation showed that the amount of collagen fibers in the anastomosis was greater in the study group than in the control group. The results suggest that the high-tension state of the digestive tract is an important factor leading to anastomotic stenosis, and thus, we put forward the Yan-Zhang’s Tissue Tension Theory of MCA to explain this phenomenon.

## Introduction

In 1978, Obora et al. performed non-suture microvascular anastomosis using magnetic rings^1^, which was the first study of cavity organ anastomosis using magnets. Subsequently, the clinician used magnetic compression anastomosis (MCA) for colorectal anastomosis^2,3^. In 1995, Cope combined MCA with endoscopic technology to achieve endoscopic gastrointestinal bypass anastomosis^4^, which has great clinical significance. MCA has been under development for over 40 years, and its research fields have expanded from the initial vascular anastomosis and gastrointestinal anastomosis to ureterovesical anastomosis^5^, biliary stricture after liver transplantation^6,7^, esophageal stricture^8^, colorectal stricture^9^, and rectovaginal fistula closure repair^10^. MCA can also be used for therapeutic purposes of vesicostomy^11^ and established pathological fistula animal models^12^.

In the development of MCA, although some scholars have initially established the staging of digestive tract MCA^13^, and found the pathological change process of magnamosis. However, compared with the more innovation in MCA technology, the research on MCA theory is still less. The anastomosis reportedly shrinks after endoscopic gastrointestinal magnetic anastomosis, and thus, it is necessary to place a gastrointestinal stent at the site of magnetic anastomosis^14,15^. After MCA of esophageal atresia or stenosis, the anastomosis will gradually become smaller, and thus, regular balloon dilation is required^16^. However, open operation MCA of the digestive tract in experimental animals has achieved good anastomosis results^17,18^.

Therefore, we speculate that the reason for this difference is the difference in tissue tension. In this study, we used Sprague–Dawley (SD) rats as experimental animals to investigate the effect of tissue tension on the size of magnetic anastomosis by preparing a gastroduodenal high-tissue tension model.

## Materials and methods

### Ethics statement

This study was reviewed and approved by the Committee for Ethics of Animal Experiments of Xi’an Jiaotong University (Permit Number: 2021-1534). All experiments were performed in accordance with relevant guidelines and regulations issued by the Xi’an Jiaotong University Medical Center for the Care and Use of Experimental Animals. Twenty SD albino rats (10 male, 10 female) weighing 200–250 g were provided by the Laboratory Animal Center of the Xi’an Jiaotong University (Xi’an China). The animals were acclimatized to laboratory conditions (23 °C, 12 h/12 h light/dark, 50% humidity, and ad libitum access to food and water) for one week before commencing the experiments.

### Study design

The 20 SD rats were randomly divided into study and control groups (n = 10 per group). To minimize the influence of surgical trauma on anastomosis formation, the parent magnet (PM) and the daughter magnet (DM) were placed into the duodenum and stomach, respectively, by transoral implantation. The gastroduodenal side-to-side anastomosis can be performed by adjusting the position of the magnets such that they attract each other. In this study, the difference between the control group and the study group was that gastroduodenal bypass magnetic anastomosis in the control group was performed in a tension-free state. Conversely, in the study group, the anastomosis was performed in a condition wherein the stomach and duodenum were inflated and the tissue was hyper-stretched. The discharge time of the magnets was recorded. Four weeks postoperatively, the rats were euthanized, and the gross specimen of the anastomosis was obtained to measure the bursting pressure of the anastomosis and the inside diameter of the anastomosis, and the healing of the anastomosis was observed by the naked eye and through light microscopy.

### Magnet

**Figure 1:**
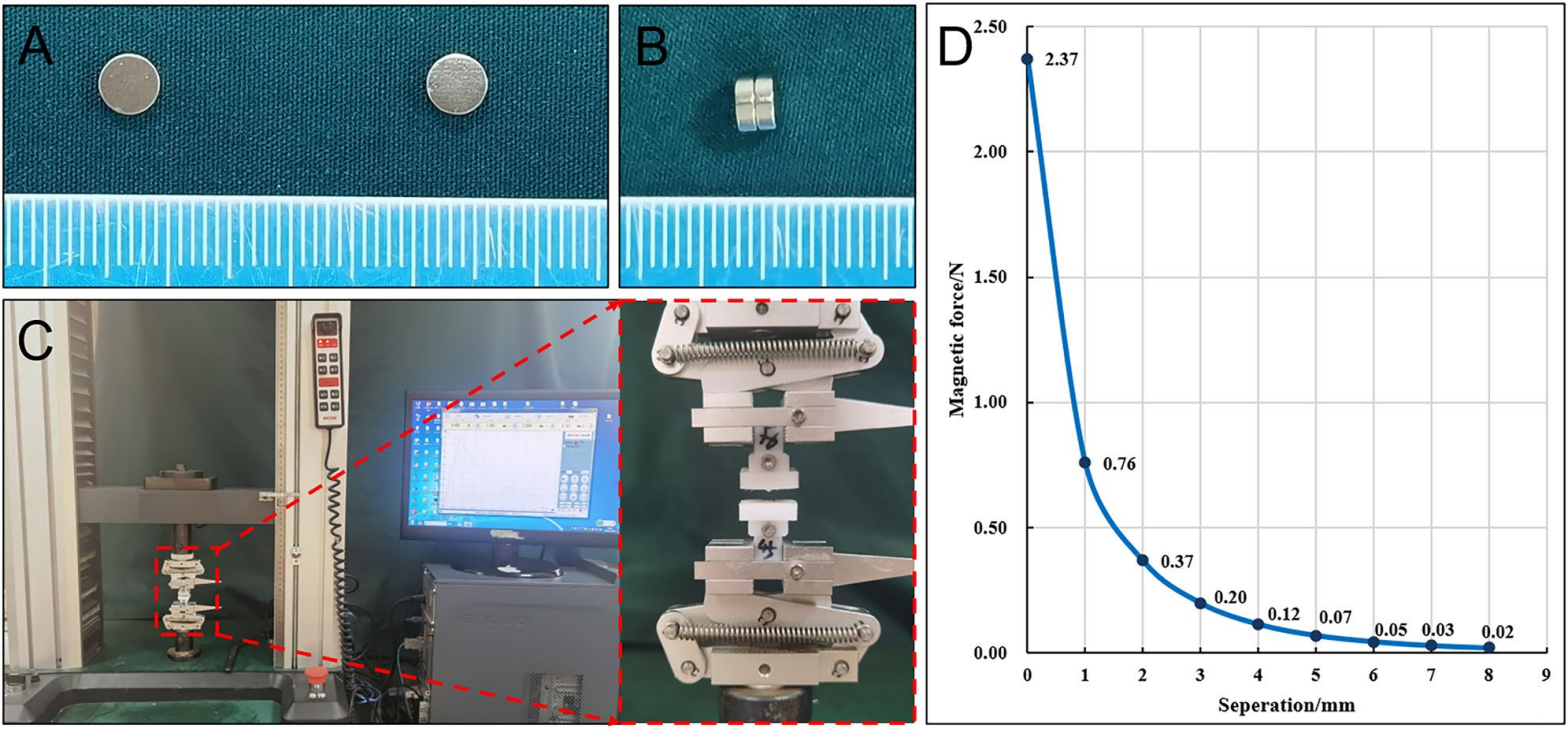
The magnet. (**A**) Frontal view of the magnets. (**B**) Side view of the magnets attracted together. (**C**) Magnetic force test platform. (**D**) Magnetic force curve.

In this study, the size and shape of the magnets used in the study and control groups were exactly the same. The magnet was a cylinder (diameter: 5 mm, height: 1.5 mm, mass: 0.23 g) with axial saturation magnetization, the working surface magnetic field intensity was 196 mT. It was made of sintered NdFeB permanent magnet material (Grade N42), and the surface of the magnet had a nickel coating (Fig. 1A,B). The magnet was manufactured by Jinshan Electronic Appliances Ltd. (Xi’an, China). The magnetic force measured by the electronic material testing machine at zero distance between two magnets was 2.37 Newtons (Fig. 1C), and the magnetic force curve of the magnets is shown in Fig. 1D.

### Surgical procedures

**Figure 2:**
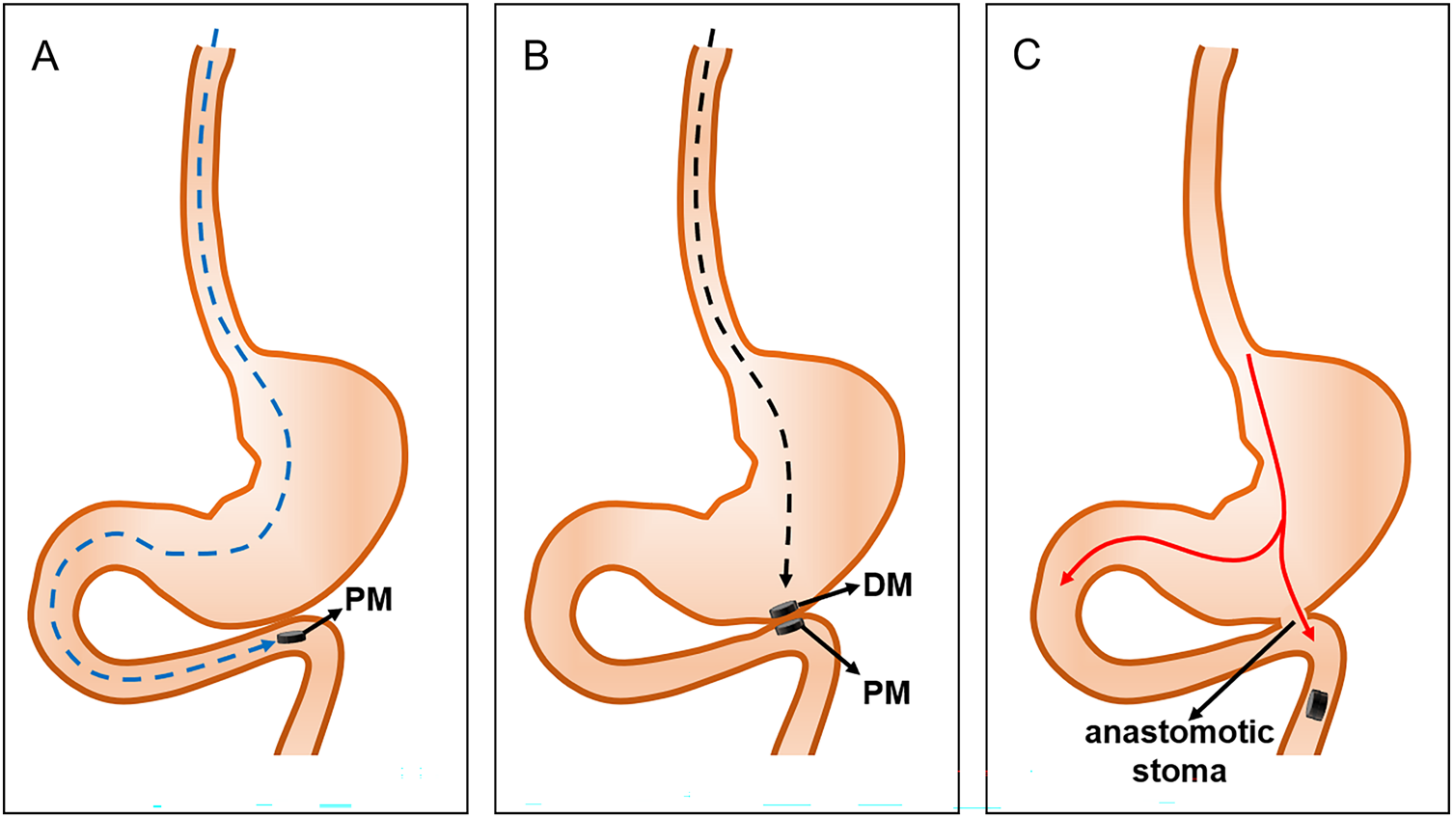
A schematic of the gastroduodenal bypass magnamosis. (**A**) The parent magnet (PM) is placed at the end of the duodenum. (**B**) The daughter magnet (DM) is placed in the stomach, and the PM and the DM are attracted together. (**C**) After the anastomosis is established, the magnets enter the distal intestine.

SD rats were intraperitoneally injected with 3% pentobarbital sodium (0.1 mL/100 g) and were fixed on the operating table in the supine position after successful anesthesia. An electric hair shaver was used to remove upper abdominal hair. The median incision of the upper abdomen was made in all the rats, which was about 3 cm long, and the stomach and duodenum were revealed after entering the abdomen. The PM was inserted through the mouth and pushed into the stomach using a plastic tube. The PM was slowly delivered to the end of the duodenum through the abdominal incision. Using the same method, the DM was inserted into the stomach through the mouth, and the position of the magnet was adjusted such that the DM and PM attracted each other to complete the gastroduodenal bypass magnetic anastomosis (Fig. 2A,B). When the magnetic anastomosis was established, the DM and PM entered the distal intestine and were eventually expelled from the body (Fig. 2C). In the study group, to keep the stomach and duodenum in a state of high tension, a plastic tube was inserted into the stomach through the mouth after the placement of the magnets, and the end of the duodenum was closed with a clamp. Then, air was slowly injected through the plastic tube to expand the stomach and duodenum, and the DM and PM were attracted under this expanded state to complete the gastroduodenal side-to-side anastomosis. The air in the stomach was pumped, and then, the plastic tube and clamp were removed. A 2–0 silken thread was used to suture the abdominal cavity.

### Postoperative care

Immediately after the operation, abdominal X-ray examination was performed to observe the status of the PM and DM. The rats had ad libitum access to food and water after awakening from anesthesia. Pethidine (0.1 mg/100 g) was administered intramuscularly every 12 h as an analgesic during the first three postoperative days. The general condition of rats was observed closely after the operation, and the discharge time of the magnets was recorded for each rat.

### Specimen collection and anastomosis measurement

All rats were euthanized 4 weeks after the operation [3% pentobarbital sodium solution (0.2 mL/100 g) intravenous rapid infusion], and gastroduodenal anastomotic specimens were obtained. The anastomosis was observed by naked eye, and the diameter of the anastomosis was measured using a conical aperture ruler.

### Anastomosis histological analysis

All anastomosis samples were immersed in 10% buffered formalin overnight. After fixation, the samples were embedded in paraffin, and 4-μm-thick sections were cut at the anastomosis site. The sections were stained with hematoxylin & eosin (HE) and Masson dye for visualization under a light microscope.

### Statistical analyses

SPSS statistics software v22.0 (IBM Corporation, Armonk, NY, United States) was used for data analysis. Quantitative data are expressed as the mean ± standard deviation (SD). Differences between the groups were compared by an independent sample t-test or a nonparametric test. *P* < 0.05 indicated a significant difference.

### ARRIVE guidelines statement

The authors have read the ARRIVE guidelines, and the manuscript was prepared and revised according to the ARRIVE guidelines.

## Results

### Procedural parameters

**Figure 3:**
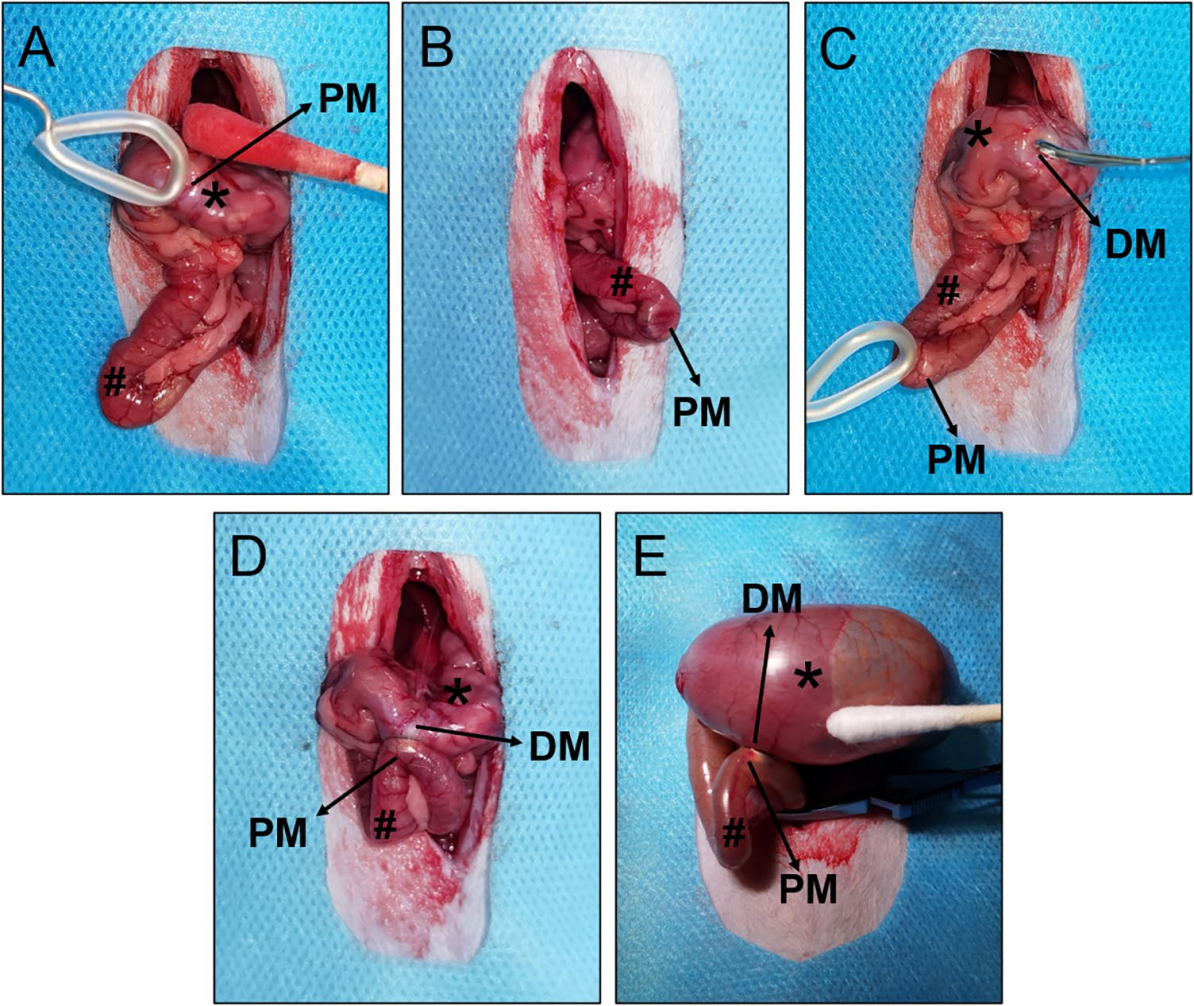
Surgical procedure. (**A**) The parent magnet (PM) reaches the stomach. (**B**) The PM reaches the end of the duodenum. (**C**) The daughter magnet (DM) reaches the stomach. (**D**) In the control group, the magnets attract each other without tension in the gastrointestinal tract. (**E**) In the study group, the magnets attract each other under high tension in the gastrointestinal tract. *stomach, ^#^duodenum.

The gastroduodenal side-to-side magnetic anastomosis was successfully performed in all rats. The placement process of both magnets was smooth; no bleeding or perforation occurred in the digestive tract, and the magnets reached the target position (Fig. 3A–C). In control and study groups, the positioning of the magnets such that they attract each other was achieved under the conditions of no tension and high tension, respectively (Fig. 3D–E). All 20 rats were simultaneously operated without any complications. There was no magnet displacement, no unintended deformation, and no digestive tract injury. The average operative time was 16.25 min ± 0.79 min (range: 15.50–17.50 min) in the control group and 20.15 ± 1.16 min (range: 18.50–22.00 min) in the study group.

### Survival rate and postoperative complications

**Figure 4:**
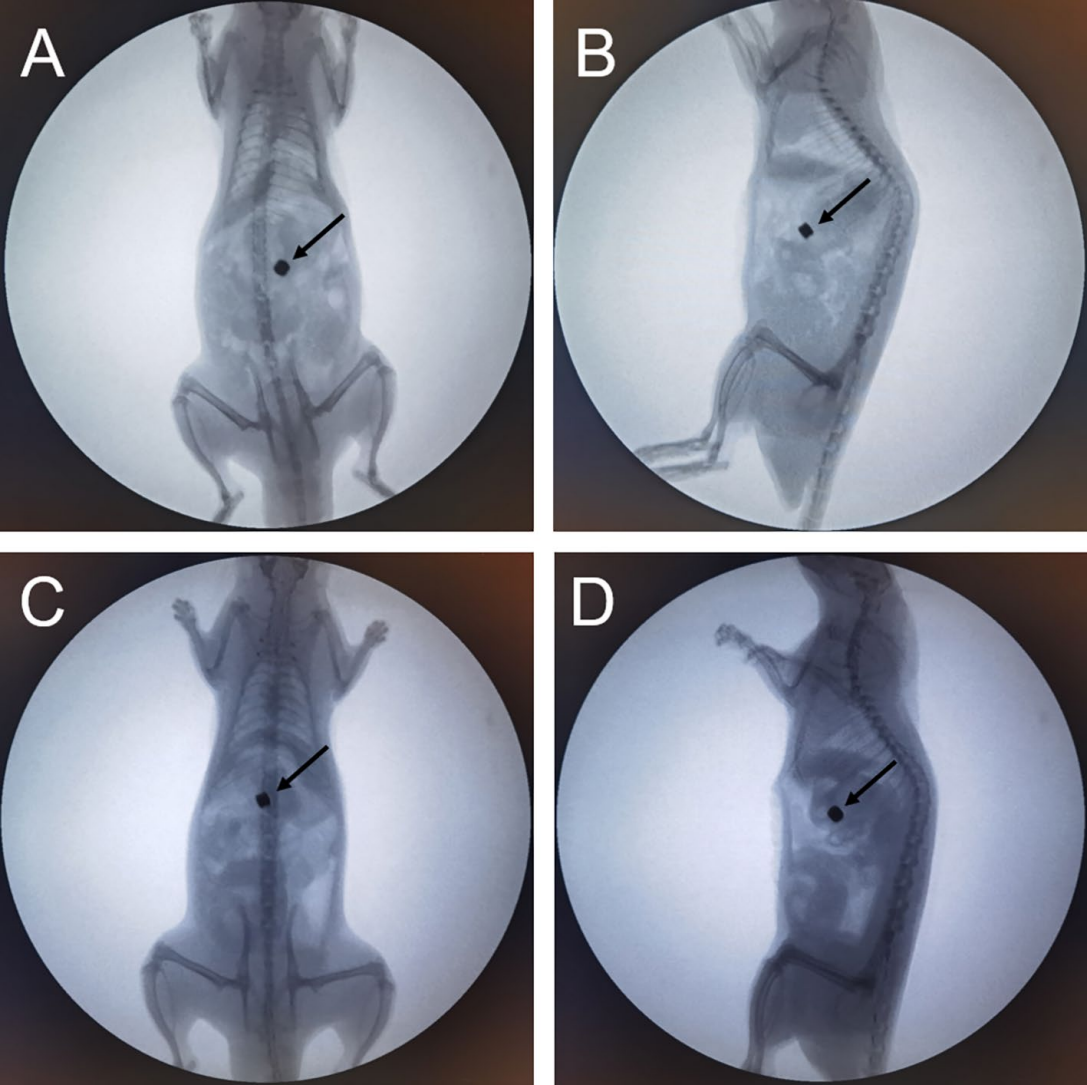
X-ray examination. (**A**) Anterior radiograph of the rat in the control group. (**B**) Lateral radiograph of the rat in the control group. (**C**) Anterior radiograph of the rat in the study group. (**D**) Lateral radiograph of the rat in the study group. The black arrows indicate magnets.

Postoperative abdominal X-ray examination indicated that the magnets were in good position (Fig. 4). All rats were generally in good health and survived until 4 weeks after surgery. At 7–10 days postoperatively, the magnets passed out of the body through the anus, and there was no evidence of intestinal obstruction.

### Gross appearance of the anastomosis

**Figure 5:**
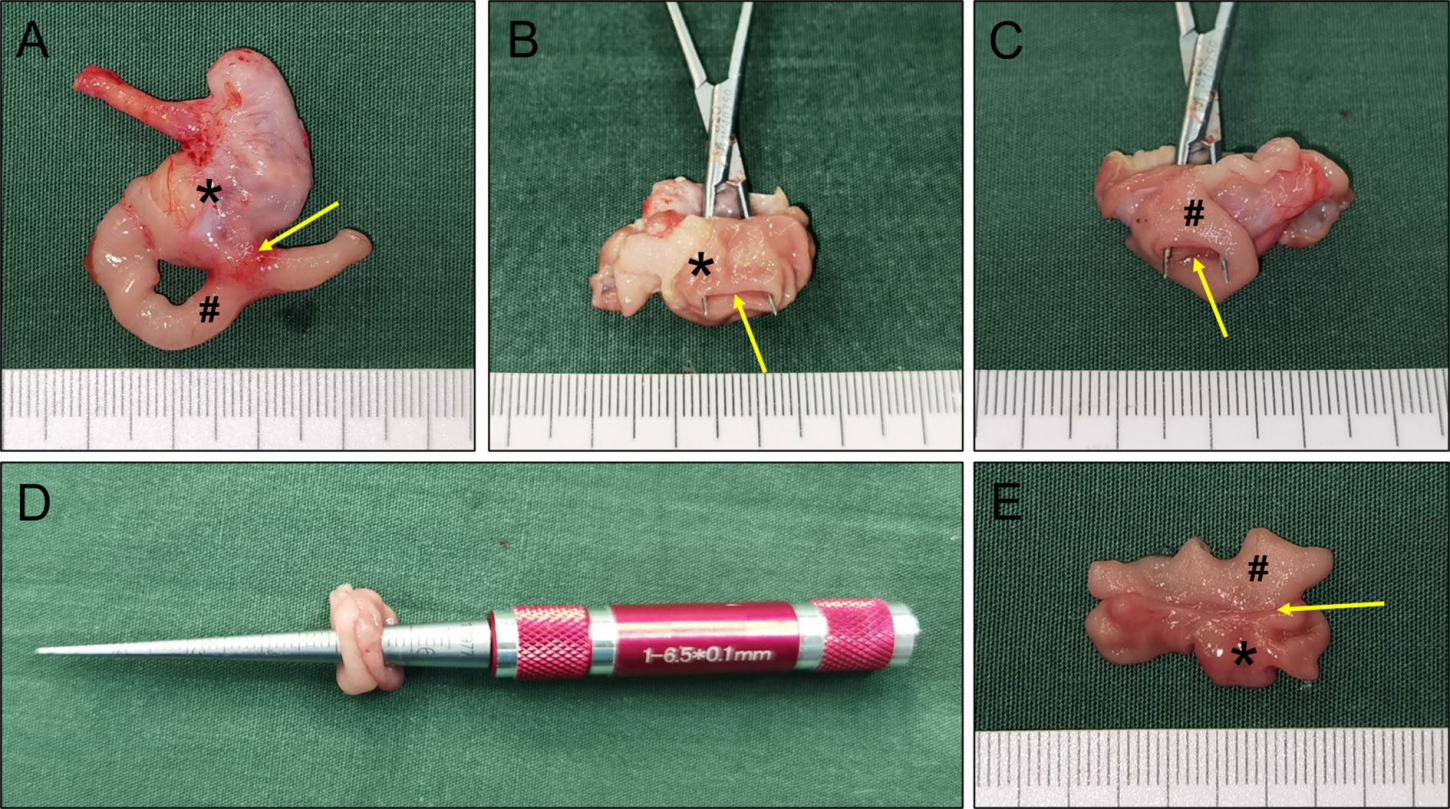
Gross specimen of the control group. (**A**) Gross appearance of the anastomosis. (**B**) The anastomosis as seen from the stomach. (**C**) The anastomosis as seen from the duodenum. (**D**) Measuring the anastomotic diameter. (**E**) The anastomotic mucosa as seen by longitudinal dissection. *stomach, ^#^duodenum. The yellow arrows indicate anastomosis.

**Figure 6:**
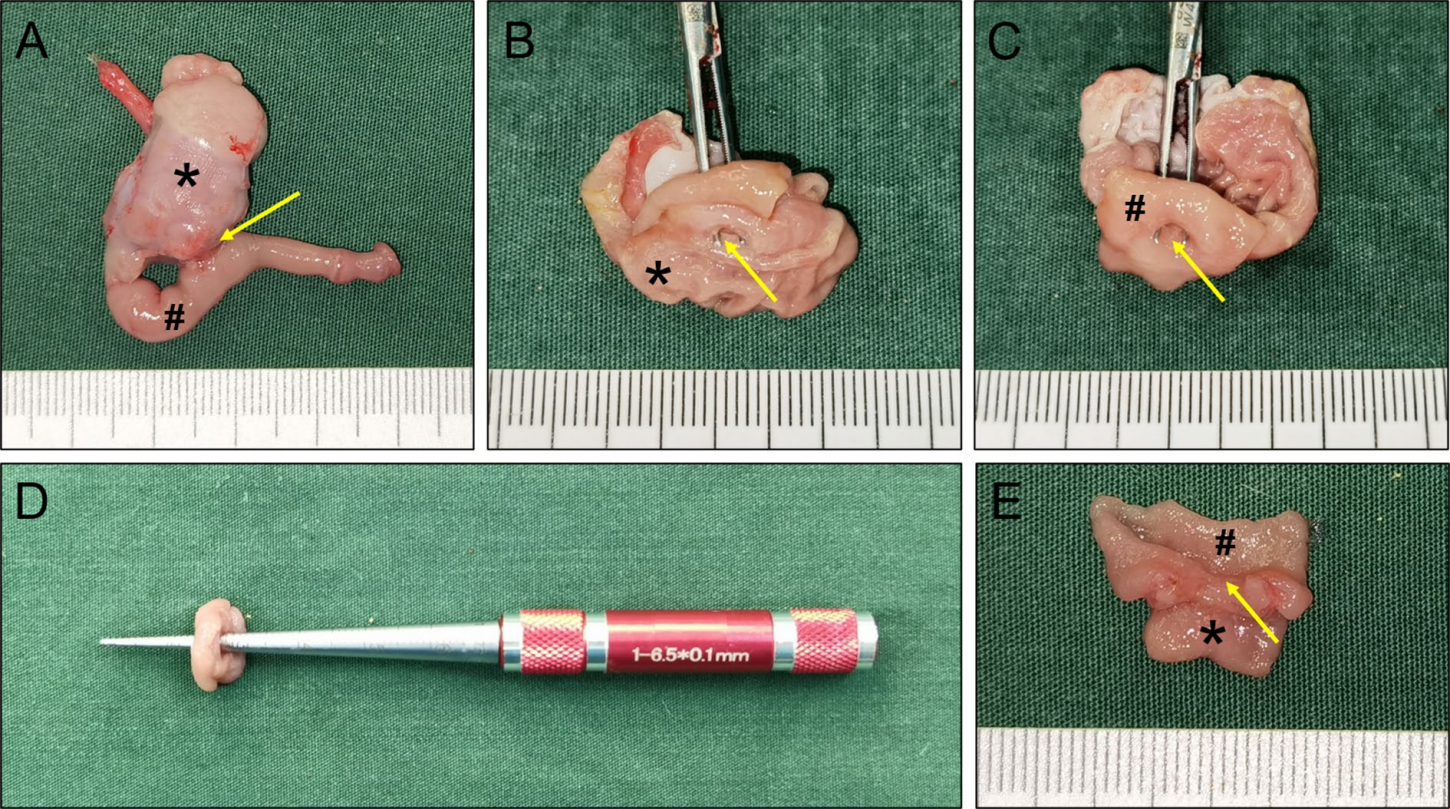
Gross specimen of the study group. (**A**) Gross appearance of the anastomosis. (**B**) The anastomosis as seen from the stomach. (**C**) The anastomosis as seen from the duodenum. (**D**) Measuring the anastomotic diameter. (**E**) The anastomotic mucosa as seen by longitudinal dissection. *stomach, ^#^duodenum. The yellow arrows indicate anastomosis.

**Figure 7:**
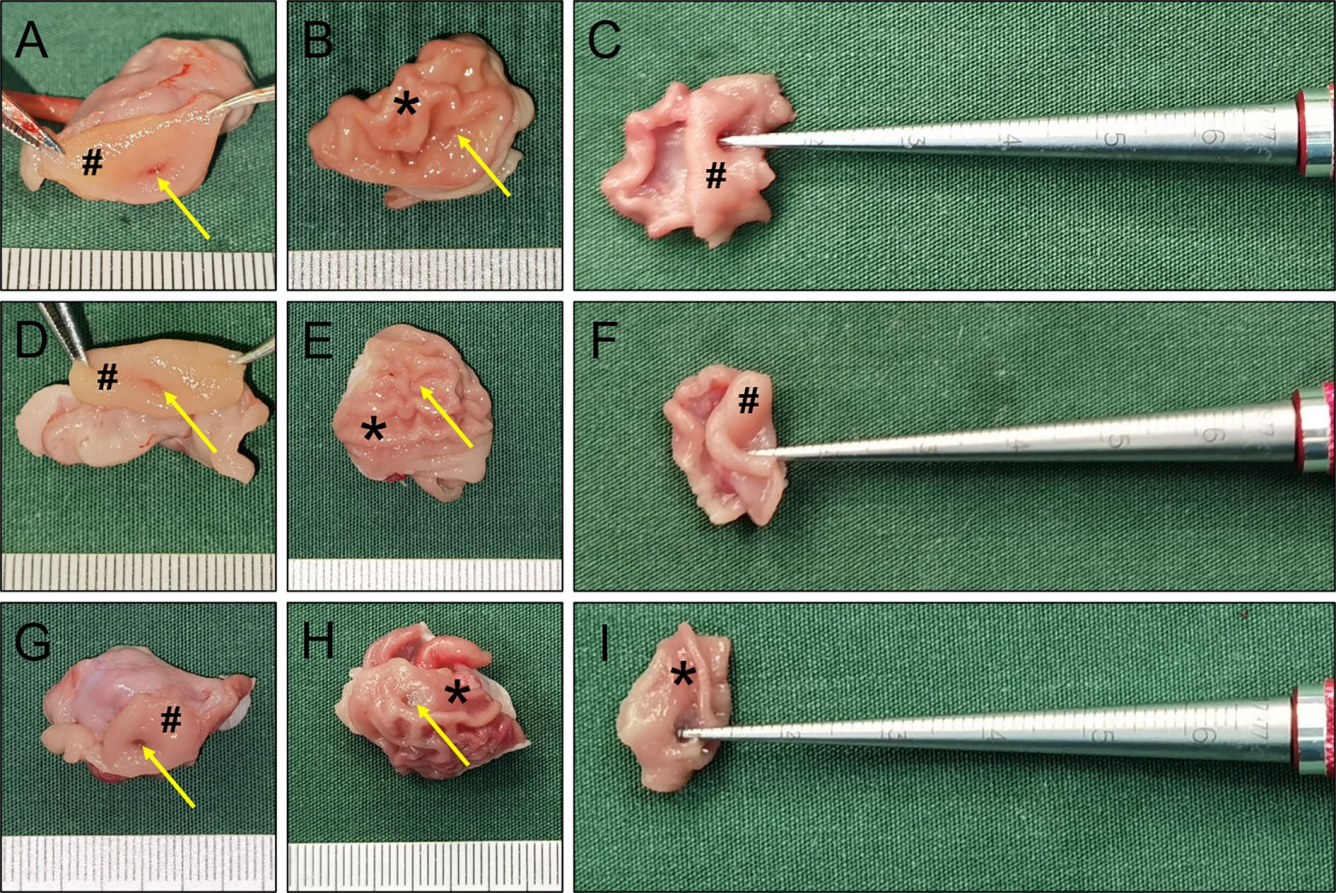
Three samples from the study group with severe anastomotic stenosis. (**A**–**C**) Gross specimen of sample 1. (**D**–**F**) Gross specimen of sample 2. (**G**–**I**) Gross specimen of sample 3. *stomach, ^#^duodenum.

Gross observation of the anastomosis specimens of the control and study groups showed that the serous surface of the gastroduodenal anastomosis was well healed, and there was no abscess or serious adhesion around the anastomosis (Figs. 5A, 6A). The mucosal surface of the anastomosis was found to be well healed upon opening the stomach and duodenum (Figs. 5B,C,E, 6B,C,E). Anastomotic diameters of both groups were measured using a conical aperture ruler (Figs. 5D, 6D). The results showed that the anastomotic diameter of the study group was significantly smaller than that of the control group (2.30 ± 1.64 mm vs. 4.78 ± 0.11 mm, *P* < 0.001). Three of the animals in the study group had nearly occlusive anastomoses (Fig. 7), which we define as severely narrow anastomosis.

### Histological observation

**Figure 8:**
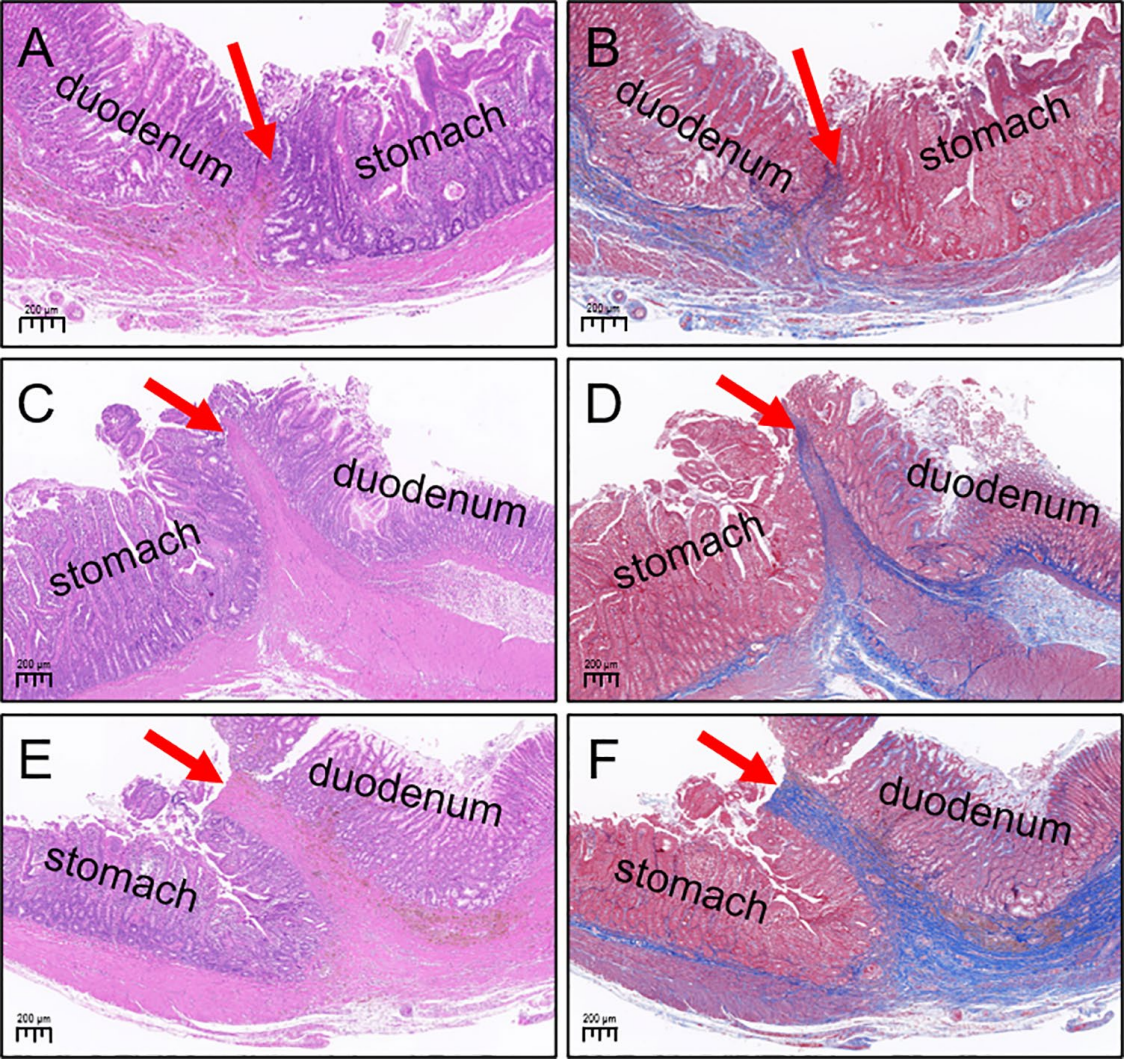
Histological observation of the anastomosis. (**A**) The anastomosis stained with HE (5.5×) in the control group. (**B**) The anastomosis stained with Masson (5.5×) in the control group. (**C**) The anastomosis stained with HE (4×) in the study group. (**D**) The anastomosis stained with Masson (4×) in the study group. (**E**) Anastomotic occluded specimens stained with HE (4×) from the study group. (**F**) Anastomotic occluded specimens stained with Masson (4×) from the study group.

HE and Masson staining of the gastroduodenal anastomosis showed good serous membrane continuity in both groups (Fig. 8A–F). Mucosal continuity was better in the control group (Fig. 8A,B), and although continuity was present in gastric and duodenal mucosa in the study group, it was slightly inferior to that in the control group (Fig. 8C,D). In the study group, HE and Masson staining showed continuity interruption of gastric mucosa and duodenal mucosa in some specimens (Fig. 8E,F), indicating occlusion of anastomosis at the histological level, and this finding was consistent with the macroscopic observation results of gross specimens. Masson staining showed that the collagen fiber content of the anastomosis was relatively low in the control group (Fig. 8B) but significantly increased in the study group (Fig. 8D). In the study group, Masson staining identified that the specimens with occluded anastomoses had significantly increased collagen fibers (Fig. 8F).

## Discussion

MCA is a new anastomosis technique; wherein non-contact magnetic force drives the establishment of endoscopic gastrointestinal anastomosis. There is an important difference between endoscopic magnetic gastroenterostomy and open magnetic gastroenterostomy. To obtain a clear field of view during endoscopic operation, gas should be continuously injected into the stomach to dilate the gastrointestinal tract. During endoscopic operation, magnetic anastomosis was completed under the condition of high tissue tension of the gastrointestinal tract. Conversely, when magnetic anastomosis was performed under laparotomy, the gastrointestinal tract was in a tension-free state. Therefore, the high tissue tension of the gastrointestinal tract may be the reason affecting the size of the anastomosis.

**Figure 9:**
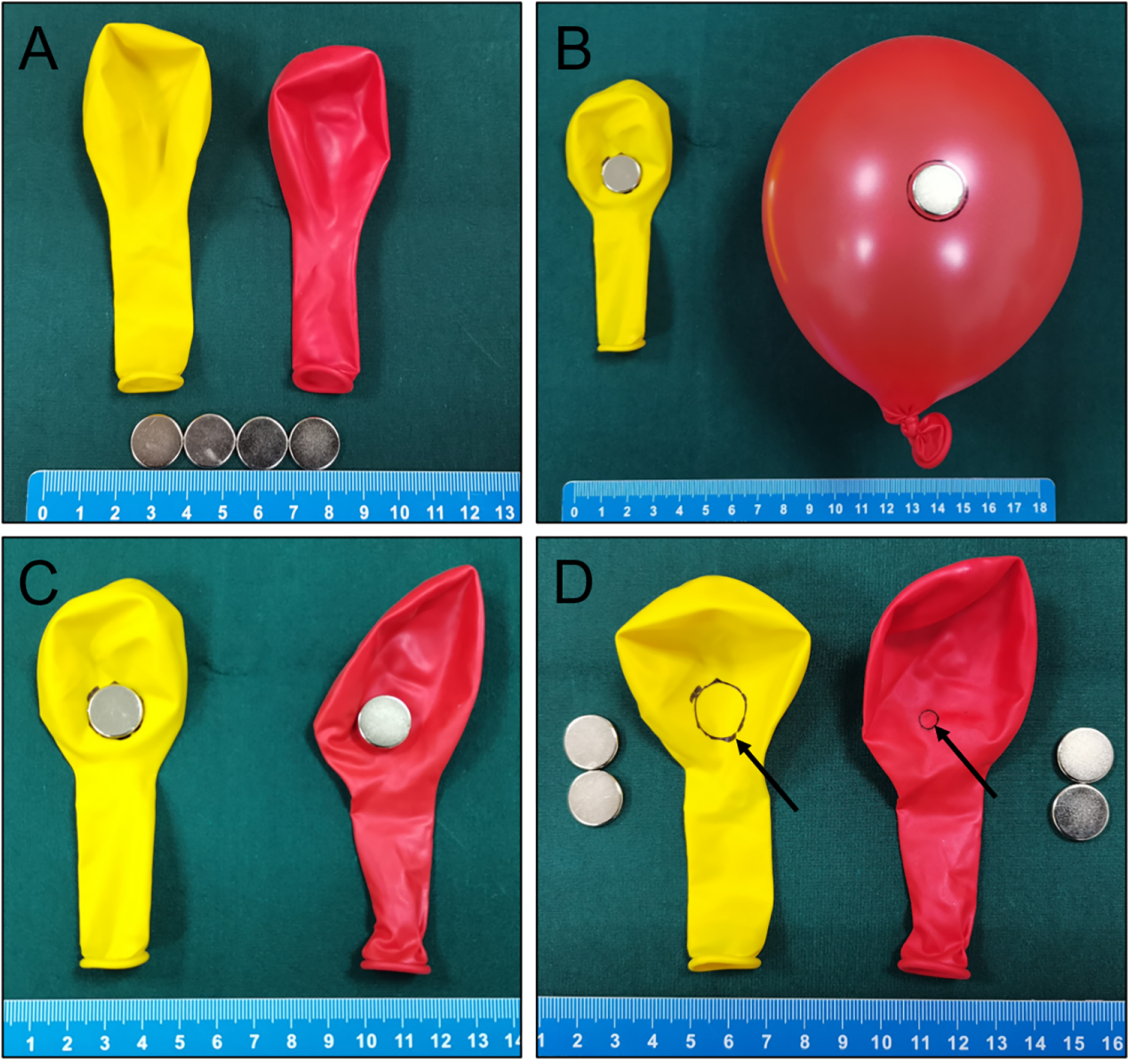
Demonstration of Yan-Zhang’s Tissue Tension Theory. (**A**) Demonstration balloons and magnets. (**B**) The magnets compressed the balloon wall in the uninflated state and inflated state, and the area of the compressed balloon wall covered by the magnet was marked using a marker pen. (**C**) The magnet position was kept unchanged, and the inflated balloon was returned to its original tension-free state. (**D**) The magnets were carefully removed, and the area of the balloon wall compressed between the two balloons was compared.

The results showed that the diameter of gastrointestinal magnetic anastomosis was significantly smaller in the study group (under high tension) than in the control group (under normal tension). These results completely support our hypothesis that the high-tension state of the gastrointestinal tissue markedly affects the size of the magnetic anastomosis. To answer why this happens, we can better understand this problem by drawing an analogy with balloons. First, we reviewed the histopathological changes caused by MCA. The compressed tissue undergoes a pathological process of ischemia, necrosis, and shedding (in that order), and the adjacent tissue is believed to undergo adherence, repair, and healing (in that order)^19^. Therefore, it is understandable that the size of the final anastomosis formed by MCA is equal to the tissue area that underwent the process of ischemia, necrosis, and shedding. Under the premise that we acknowledge this point, we attempted to exemplify the above phenomena with two identical balloons (Fig. 9A). One balloon was in an inflated state (high-tension state) after being injected with a large amount of air, and the other balloon was uninflated (tension-free state). These two balloons represent the stomach under gastroscopy and the stomach under laparotomy, respectively. Two magnets of the same diameter were used to compress the walls of both balloons, one in the high-tension state and the other in the non-tension state (Fig. 9B), and the edges of the magnet were marked over the balloon using a marker pen. The air was then released to return the balloon to a tension-free state (Fig. 9C). Upon removing the magnet compressed against the balloon wall, it could be clearly seen that the area of the balloon wall sandwiched between the two magnets was not equal in the two balloons (Fig. 9D). Through this simulation experiment, we can understand why gastrointestinal anastomosis is more prone to stenosis under gastroscopy. We call this the Yan-Zhang’s Tissue Tension Theory of MCA. Specifically, the digestive tract in the state of high tension is like an inflated balloon. The increase in the surface area upon stretching reduces the real tissue area compressed by the magnets, resulting in the diameter of the anastomosis established by the magnets being much smaller than the diameter of the magnets.

Many factors affect the establishment of MCA. We propose that the Yan-Zhang’s Tissue Tension Theory of MCA is not to deny the role of other factors but to supplement these factors. However, it is undeniable that this theory can well explain why the anastomosis becomes smaller after endoscopic gastrointestinal MCA.

To our knowledge, this study is the first to propose that tissue tension can affect the establishment of MCA of the digestive tract using animal experiments, which is a major theoretical breakthrough for MCA and has important significance. Nevertheless, this study has some limitations. First, more convincing conclusions could have been obtained if experimental dogs or pigs had been used to complete the entire MCA operation under gastroscopy because their anatomy and tissue structure are more similar to those of humans. Second, the observation time after the establishment of anastomosis was short, and analysis of the anastomosis specimens of more than 6 months would have yielded significant insight and findings. Third, this study is qualitative, and it will be of greater value if the functional relationship between tissue tension and anastomotic size can be further determined.

## Conclusion

In conclusion, the tissue tension of digestive tract can affect the size of magnetic anastomosis. The high-tension state is not conducive to establishing an unobstructed magnetic anastomosis. The Yan-Zhang’s Tissue Tension Theory can scientifically explain why endoscopic gastrointestinal MCA is more prone to stenosis.

## Data Availability

The datasets used and analyzed during the current study available from the corresponding author on reasonable request.

## References

[1] Obora Y, Tamaki N, Matsumoto S (1978). Nonsuture microvascular anastomosis using magnet rings: Preliminary report. Surg. Neurol..

[2] Jansen A, Keeman JN, Davies GA, Klopper PJ (1980). Early experiences with magnetic rings in resection of the distal colon. Neth. J. Surg..

[3] Jansen A, Brummelkamp WH, Davies GA, Klopper PJ, Keeman JN (1981). Clinical applications of magnetic rings in colorectal anastomosis. Surg. Gynecol. Obstet..

[4] Cope C (1995). Creation of compression gastroenterostomy by means of the oral, percutaneous, or surgical introduction of magnets: Feasibility study in swine. J. Vasc. Interv. Radiol..

[5] An Y (2023). An experimental study of magnetic compression technique for ureterovesical anastomosis in rabbits. Sci. Rep..

[6] Jang SI, Cho JH, Lee DK (2020). Magnetic compression anastomosis for the treatment of post-transplant biliary stricture. Clin. Endosc..

[7] Nakaseko Y (2017). Successful treatment of stricture of duct-to-duct biliary anastomosis after living-donor liver transplantation of the left lobe: A case report. Transpl. Proc..

[8] Zhang M (2023). A novel deformable self-assembled magnetic anastomosis ring (DSAMAR) for esophageal stenosis recanalization without temporary gastrostomy in beagle dogs. J. Pediatr. Surg..

[9] Zhang M (2023). A novel self-shaping magnetic compression anastomosis ring for treatment of colonic stenosis. Endoscopy.

[10] Yan XP (2016). Magnet compression technique: A novel method for rectovaginal fistula repair. Int. J. Colorectal Dis..

[11] Zhang M (2022). A novel magnetic compression technique for cystostomy in rabbits. Sci. Rep..

[12] Gao Y, Wu RQ, Lv Y, Yan XP (2019). Novel magnetic compression technique for establishment of a canine model of tracheoesophageal fistula. World J. Gastroenterol..

[13] Zhang M (2022). Establishment of Yan-Zhang's staging of digestive tract magnetic compression anastomosis in a rat model. Sci. Rep..

[14] Cope C, Clark TW, Ginsberg G, Habecker P (1999). Stent placement of gastroenteric anastomoses formed by magnetic compression. J. Vasc. Interv. Radiol..

[15] Cope C, Ginsberg GG (2001). Long-term patency of experimental magnetic compression gastroenteric anastomoses achieved with covered stents. Gastrointest. Endosc..

[16] Zaritzky M, Ben R, Johnston K (2014). Magnetic gastrointestinal anastomosis in pediatric patients. J. Pediatr. Surg..

[17] An Y (2018). Gastrojejunal anastomosis in rats using the magnetic compression technique. Sci. Rep..

[18] Zhang M (2022). Magnetic compression technique for esophageal anastomosis in rats. J. Surg. Res..

[19] Yan XP (2019). Exploration and establishment of magnetic surgery. Chin. Sci. Bull..

